# Development of Multifunctional Biopolymeric Auto-Fluorescent Micro- and Nanogels as a Platform for Biomedical Applications

**DOI:** 10.3389/fbioe.2020.00315

**Published:** 2020-04-30

**Authors:** Arti Vashist, Venkata Atluri, Andrea Raymond, Ajeet Kaushik, Tiyash Parira, Zaohua Huang, Andriy Durygin, Asahi Tomitaka, Roozbeh Nikkhah-Moshaie, Atul Vashist, Marisela Agudelo, Hitendra S. Chand, Ilyas Saytashev, Jessica C. Ramella-Roman, Madhavan Nair

**Affiliations:** ^1^Department of Immunology and Nanomedicine, Center for Personalized Nanomedicine, Herbert Wertheim College of Medicine, Institute of NeuroImmune Pharmacology, Florida International University, Miami, FL, United States; ^2^Division of Sciences, Art, and Sciences, Department of Natural Sciences, Florida Polytechnic University, Lakeland, FL, United States; ^3^Department of Otolaryngology, University of Miami School of Medicine, Miami, FL, United States; ^4^CeSMEC, Florida International University, Miami, FL, United States; ^5^Department of Biotechnology, All India Institute of Medical Science, New Delhi, India; ^6^Department of Biomedical Engineering, Florida International University, Miami, FL, United States; ^7^Department of Cellular Biology, Pharmacology and Ophthalmology, Herbert Wertheim College of Medicine, Miami, FL, United States

**Keywords:** nanogels, microgels, theranostics, nanomedicine, biopolymers

## Abstract

The emerging field of theranostics for advanced healthcare has raised the demand for effective and safe delivery systems consisting of therapeutics and diagnostics agents in a single monarchy. This requires the development of multi-functional bio-polymeric systems for efficient image-guided therapeutics. This study reports the development of size-controlled (micro-to-nano) auto-fluorescent biopolymeric hydrogel particles of chitosan and hydroxyethyl cellulose (HEC) synthesized using water-in-oil emulsion polymerization technique. Sustainable resource linseed oil-based polyol is introduced as an element of hydrophobicity with an aim to facilitate their ability to traverse the blood-brain barrier (BBB). These nanogels are demonstrated to have salient features such as biocompatibility, stability, high cellular uptake by a variety of host cells, and ability to transmigrate across an *in vitro* BBB model. Interestingly, these unique nanogel particles exhibited auto-fluorescence at a wide range of wavelengths 450–780 nm on excitation at 405 nm whereas excitation at 710 nm gives emission at 810 nm. In conclusion, this study proposes the developed bio-polymeric fluorescent micro- and nano- gels as a potential theranostic tool for central nervous system (CNS) drug delivery and image-guided therapy.

## Introduction

Numerous systems have been developed as theranostics agents, which included multi-functional inorganic nanoparticles (Degli Esposti et al., 2018), quantum dots (Ho and Leong, 2010), and many radiolabeled biomarkers (Kang et al., 2018), however, the clinical applications of these agents have provoked practical challenges. Recently, theranostics have been tuned as a personalized health-care due to advancements in drug delivery systems (DDS) for enhanced efficacy and least side-effects. The current strategy of developing bio-polymeric theranostic agents has pronounced conviction of incorporating inherent features of no toxicity, biodegradability, biocompatibility and high sensitivity.

In this regard, fluorescent hydrogels have attracted great attention of us and others due to their smart biomaterials like features and have been intensively explored as most convenient tracers and therapeutics for decades. Many studies showed the development of fluorescence-based hydrogels for sensing and tracking the bio-actives and therapeutics *in vitro and in vivo* (Pires et al., 2018). However for these diverse applications, hydrogels need to be developed by tagging the fluorophores by chemical or physical immobilization inside the hydrogel matrix. The entrapment of fluorophores in the hydrogel matrix is tedious and limiting process as the tagging or entrapping fluorescent dyes or compounds and their photobleaching may affect their characteristic features like biocompatibility and biodegradability (Zhang and Yang, 2013). Such DDS has exhibited performance in a personalized manner due to tunable salient features including size, morphology, targeted delivery, and release profile (Allen and Cullis, 2004).

Efforts have been made to develop macro to micro- and nano-size hydrogel particles, exhibiting lower toxicity. Literature reveals both micro and nano range is preferred for various cancer therapies and other biomedical applications (Sahiner et al., 2006; Zhang et al., 2015; Vashist et al., 2018a). Thus, in the present study, the focus was more on the development of bio-polymeric hydrogel particles in both microscale and nanoscale, which have the following features: (i) easy synthesis with high yield; (ii) highly biocompatible to the intracellular environment; (iii) the by-products of the hydrogels are biodegradable and non-toxic to the cellular environment; (iv) have functionality which makes them capable of binding with various bio-actives including drugs, DNA, RNA, proteins, etc.; (v) the biomaterials can be detected *in vitro and in vivo*; and (vi) the cellular uptake and the monitoring or tracking of the carrier for release and degradability should be feasible (Shi et al., 2014). The combination of the above features makes nanogels an excellent drug carrier with extraordinary performance in drug delivery and diagnostics (Hamidi et al., 2008; Jiang et al., 2014; Vashist et al., 2017).

Efforts have been made to develop nanogel based systems as carriers for those drugs that cannot pass through the BBB. Therefore, efficient nanogel carriers have been developed to deliver hydrophobic drugs, oligonucleotides, and other bio-actives across the BBB (Vinogradov et al., 2004; Roney et al., 2005; Juillerat-Jeanneret, 2008). Various cells like pericytes, astrocytes, and endothelial cells constitute BBB. The structure and the functionality of the BBB are related to the tightly-packed endothelial cells. Existing therapies for CNS are defeated by the challenges imposed by the BBB that hinders the entry of several drugs and bioactive across it. Strategies to develop nanoparticles with the potential to traverse the BBB by changing their surface functionality or by the widespread mechanism “Trojan horse,” which relates to the engulfment of nanoparticles by the endogenous transport systems (Wohlfart et al., 2012; Wong et al., 2012). The systems which involve inorganic particles and synthetic polymers, are associated with certain drawbacks such as biodegradability, toxicity and other side effects (Hamid Akash et al., 2015). Thus, there is an immense need to develop biodegradable and biocompatible cost-effective DDS. The goal of the present study was to achieve a very simple and stable bio-polymeric hydrogel system, which can be sorted to various sizes for diverse biomedical applications. The developed natural polymer based hydrogel particles are expected to hold potential to deliver various bioactives such as drugs and proteins across the BBB.

It was anticipated that the developed particles when hydrophobically modified using linseed oil-based polyol will develop a surface functionality, which will facilitate them to cross BBB and their entry to the brain through the tight junctions. The synthesis was inspired by the fact that the synergism achieved by the combination of the two biopolymers chitosan and HEC with polyol will result in excellent drug delivery platform whose characteristics can be modulated by imbibing various bioactive or drugs for targeted and sustained delivery. We were able to develop a multi-functional material exhibiting an excellent wide excitation/emission spectrum. For the first time, we demonstrate the development of bio-polymeric auto-fluorescent hydrogels in both micro and nanoscale using natural polymers chitosan, HEC and sustainable linseed oil-based polyol exhibiting complete biocompatibility (in the concentration range 10–100 μg/ml) tested over a wide range of host cells like Astrocytes, PBMCs, and Microglia. Moreover, a dynamic wide range of emission wavelength (450–750 nm), and (710–810 nm) adds to their utility for *in vivo* imaging. The high stability in aqueous solution and physiological pH (water, pH 7.4) contributed to a good shelf-life in solution and dry form at room temperature for 12 months while retaining their auto-fluorescence property. Wide range of size from microscale to nanoscale resulted in cellular uptake and co-localization during *ex vivo* studies with PBMC, Microglia, and Astrocytes. Remarkably, the developed hydrogel particles showed the capability to transmigrate BBB, which highlights their huge potential to be used for the drug delivery to the CNS. These micro- and nanogels have unique and superior properties as compared to the existing theranostic systems (Kunjachan et al., 2012) and thus hold potential for multiple applications including drug delivery, diagnostics, and *in vivo* imaging.

## Experimental Section

### Materials

Chitosan (448877-50G, Sigma-Aldrich), Hydroxyethyl cellulose (22–300 mPa.s, 2% in water at 20°C, TCI, 9004-62-0), Heavy liquid paraffin oil: Density: 0.8660–0.890 Kg/m^3^, Tween 80, Ethanol) n-Hexane (Sigma-Aldrich), Glycine (Mwt. 75.0 7g/mol: Density 1.607 g/cm^3^, linseed oil, glacial acetic acids, hydrogen peroxide, diethyl ether, acetic anhydride (Sigma-Aldrich) were used as received. Linseed oil polyol was prepared using standard protocols (Sharmin et al., 2007). Cellulase, Trichoderma viride, Millipore Sigma Deionized water from Millipore mille U10 water purification system was used in the preparation of hydrogels and other *in vitro* experiments.

### Methods

#### Synthesis of Micro/Nano Hydrophobically Modified Chitosan-Hydroxyethyl Cellulose

The micro/nano hydrogel particles of chitosan and hydroxyethyl cellulose (HEC) were prepared by water in oil emulsion polymerization method (Kajjari et al., 2011). Linseed oil-based polyol was used as a hydrophobic modifier (Vashist et al., 2012, 2013). Forty milliliter of 2% (w/v) polymer solution was prepared by using a known amount of chitosan and HEC in 1% (v/v) acetic acid. The different formulation was prepared using medium molecular weight and low molecular weight chitosan. A separate beaker was used to make a mixture of liquid paraffin oil and 1% (w/w) Tween 80. The polymer solution was added dropwise to the mixture of oil and surfactant with a stirring rate of 1400 rpm on a magnetic stirrer. The mixing of the solution was continued for a further 20 min and followed by the addition of glutaraldehyde (5 ml) for another 10 min. The linseed oil polyol was added to the reaction mixture and stirring was continued at 1400 rpm for 6 h. The synthesized hydrogel particles were washed with n-hexane to remove the excess of oil. The excess amount of GA was deactivated by 0.1 M glycine solution (Kajjari et al., 2011). The hydrogel particles were dried for 24 h at room temperature. The throughly washed particles were kept for drying at room temperature, stored in a vaccum dessicator and used for further characterizations.

#### Characterization of Micro/Nanogels Using FT-IR, Raman, DLS, Zeta Potential, and TEM Analysis

The hydrogel samples were dried under vacuum for overnight till attained the constant weights. The dried samples were then analyzed using model 1750 FT-IR spectrophotometer (PerkinElmer Cetus Instruments, Norwalk, CT, United States). TEM analysis was performed using a Phillips CM-200 200 kV transmission electron microscope with an operating voltage of 80 kV. Particle size distribution of microgels and nanogels particles in PBS was measured by dynamic light scattering (DLS) method using a zetrasizer nano ZS (Malvern Instruments, United Kingdom) The surface charge of the particles was also determined using Zetasizer nanoZS. The hydrogel particle suspension diluted with PBS (0.1 mg/ml) was further used for both particle size and zeta potential measurements.

#### Cellular Uptake of Nanogels by Human Peripheral Blood Mononuclear Cells (PBMC), Microglial Cell Lines (CHME5), Primary Human Astrocytes, and Primary Human Microglia

##### PBMCs.

PBMCs were purified from human leukopacks (buffy coat), which were obtained commercially from the community blood bank (One Blood, Miami, FL, United States). PBMCs were isolated as previously described by us (Atluri et al., 2016). The first step involved the dilution of the buffy coat with phosphate buffer saline (PBS) (Invitrogen, Gaithersburg, MD, United States) at room temperature. The diluted buffy coat was overlaid on the top of the Ficoll-Histopaque such as two separate layers of the liquid is formed. These samples were centrifuged at 1,200 g for about 20 min with acceleration = 1, deceleration = 0 at room temperature. A further collection of the PBMC layer formed at the interface was done and the cells were washed with PBS. The pellet was re-suspended in Ammonium-Chloride-Potassium (ACK) lysing buffer to achieve complete lysis of the red blood cells in the samples and kept in ice for 15 min. The washing of the cells with PBS was again done and the total cell number and cell viability were evaluated by trypan blue exclusion (Sigma-Aldrich, St. Louis, MO, United States) in a hemocytometer counting chamber. Finally, the cells were re-suspended in complete culture medium containing Roswell Park Memorial Institute (RPMI) 1640 (Life Technologies, Gaithersburg, MD, United States), 25 mM 4-(2-hydroxyethyl)-1-piperazineethanesulfonic acid (HEPES) (Sigma-Aldrich, St. Louis, MO, United States), 2 mM glutamine (Sigma-Aldrich, St. Louis, MO, United States), 100 μg streptomycin (Sigma-Aldrich, St. Louis, MO, United States), 100 U penicillin (Sigma-Aldrich, St. Louis, MO, United States), and 10% fetal bovine serum (Life Technologies, Gaithersburg, MD, United States) (Figueroa et al., 2016).

##### CHME5.

For the biocompatibility and uptake study, the microglial cells (CHME-5) were cultured using Dulbecco’s Modified Eagle Medium (DMEM) supplemented by a fetal bovine serum (FBS) and antibiotic/antimycotic solution to a final concentration of 500 ml of DMEM + 50 ml of 10% FBS + 5.5 ml of 10X antibiotic/antimycotic solution (Sigma-Aldrich, St. Louis, MO, United States) (Samikkannu et al., 2016).

##### Primary human astrocytes.

Primary human astrocytes were purchased from ScienCell Research Laboratories (Carlsbad, CA; Cat. # 1800-5). These cells were grown on the astrocyte medium purchased from ScienCell laboratories (Cat. # 1801) containing 2% of fetal bovine serum (ScienCell Cat. # 0010), astrocyte growth supplement (ScienCell Cat. # 1852) and penicillin/streptomycin (ScienCell Cat. # 0503), antibiotic/antimycotic solution (Sigma-Aldrich, St. Louis, MO, United States) (Atluri et al., 2013).

#### Biocompatibility Analysis Using Primary Human Astrocytes, Microglia (CHME5), and PBMCs

Biocompatibility was assessed using XTT cell viability assay, using sodium 3,3′-(-[(phenylamino)carbonyl]-3,4-tetrazolium)- bis(4-methoxyl-6-nitro)benzene sulfonic acid hydrate) assay. Primary human astrocytes (1 × 10^4^ cells per well) were seeded in a 24-well plate and after 24 h of incubation at 37°C, the medium was replaced with 1 ml of fresh medium containing nanogel 5–100 μg/ml. Cells were treated with various concentrations and incubated for different time points (1, 2, 4, and/or 7 days). XTT 1 mg/ml and 2.5 μl of phenazine methosulfate (PMS) solution was freshly prepared and added (25 μl) to each well. The XTT containing wells were incubated for 4 h at 37°C. A multi-mode microplate reader (Synergy HT), was used to measure absorbance at 450 nm wavelength. All experiments were performed in triplicates (*N* = 3). Results are graphed as mean ± standard deviation. The statistical analysis was done using Two-way analysis of variance (ANOVA) and also by Tukey’s multiple comparison test. Differences were considered significant if *p* ≤ 0.05. For experiments with CHME5 and PBMCs, 2 × 10^5^ cells were seeded per well (2 × 10^5^ per well) in 24-well plates. Further, the same protocol described above was followed for each cell type. To maintain the PBMCs for 7 days, fresh media containing IL-2 was added at required intervals.

#### Determination of Lactate Dehydrogenase (LDH) Cytotoxicity of Nanogel in Microglia (CHME5) and PBMCs

CHME5 cells and PBMCs (10,000 cells per well) were plated in a 96-well plate and incubated at 37°C, 5% CO_2_. Various concentrations (10–100 μg/ml) of nanogel formulations were added to the culture media and incubated for 24 h. The Thermo Scientific Pierce LDH cytotoxicity assay kit was used to quantitatively measure lactate dehydrogenase (LDH) released into the media from damaged cells, which act as a biomarker for the cellular cytotoxicity and cytolysis (Decker and Lohmann-Matthes, 1988; Minaeva et al., 2017; Kumar et al., 2018). LDH background activity was determined by including, complete medium control. Additional cells were plated in triplicate for spontaneous LDH activity controls (negative control with water) and maximum LDH activity controls (positive control with 10X lysis buffer). Plates were incubated overnight in a CO2 at 37°C. The cells were treated for 24 h with (10–100 μg/ml) nanogel formulations. Further the Lysis Buffer (10 μl, 10X) was added to the wells serving as maximum LDH activity controls and mixed gently by tapping. Further, the plate was incubated in an incubator at 37°C, 5% CO_2_ for 45 min. Fifty microliter of medium from each sample medium (e.g., complete medium, serum-free medium, spontaneous LDH activity controls, compound-treated, and maximum LDH activity controls) was transferred to a 96-well flat-bottom plate in triplicate wells using a multichannel pipette. Further 50 μl of reaction mixture was added to each well and mixed using multichannel pipette. The plate was incubated at room temperature for 30 min in dark. After 30 min of incubation, stop solution (50 μl) was added to each sample well and mixed gently by tapping. Absorbance was measured at 490 and 680 nm. Determination of LDH activity was done by subtracting the 680 nm absorbance value of (background) from the 490 nm absorbance before calculating the percentage of cytotoxicity (Brown et al., 2015) with the following formula:

$$\%\text{Cytotoxicity=}\frac{\text{Compound}\;\text{treated}\;\text{LDH}\;\text{activity}\;-\;\text{Spontaneous}\;\text{LDH}\;\text{activity}}{\text{Maximum LDH activity}\;-\;\text{Spontaneous LDH activity}}\text{X100}$$

#### Cellular Uptake of Nanogels by Imaging Flow Cytometry

Prior to analyzing cellular uptake, we analyzed the fluorescent properties of nanogels without cells to determine the optimum concentration and maximum fluorescent intensity. Since these nanogels have multi-fluorescent properties and in this FlowSight instrument they are detected through multiple channels, a screening of different concentrations of particles (1–100 μg/ml) was done prior to selecting the optimum concentration and channel use for further analysis. 50 μg/ml of nanogels had the maximum fluorescent intensity detected through channel 8 (ex/em: 405 nm/505–560 nm). Therefore, channel 8 was selected for subsequent analysis.

To determine time-dependent uptake of nanogels, PBMCs and CHME5 (1 × 10^6^) were incubated for up to 24 h with different concentrations (1–100 μg/ml) of nanogels. Cells were harvested at different time points (2, 6, and 24 h) and washed prior to acquisition. Fluorescence intensity and percentage of cells expressing nanogels were analyzed using imaging flow cytometry with Amnis FlowSight instrument (Luminex Corporation). A total of 10,000 events were collected for all individual samples. Analysis was done using Ideas Software.

For all experiments, cells were analyzed and selected based on higher gradient RMS values or cells with better focus and single cells. Gating of focus cells is depicted in histogram (Supplementary Figure S5). After focus cells were selected, a scatter plot of brightfield area versus aspect ratio for the focus population was further analyzed to select single cells (Supplementary Figure S5), which are characterized by an intermediate area value and a high aspect ratio. The subpopulation of single cells was used to gate based on fluorescent intensity. Region on histogram was drawn based on comparisons between cells without nanogels (control sample) and cells with different concentrations of nanogels. Time-dependent cellular uptake of nanogels for 2 and 6 h is demonstrated in bar graphs included in Supplementary Figure S5. Representative histograms showing subpopulation of cells with high fluorecent intensity (MFI) and expressing nanogels (% gated) are depicted in Supplementary Figure S5.

#### Two-Photon Imaging

1 × 10^6^ cells were plated on a 2-chamber slide until cells reached about 80% confluency. The cells were treated with 50 μg/ml of nanogel concentration and incubated for different time points 6 and 24 h at 37°C, 5% CO_2_. The control and nanogel treated cells were washed thoroughly using PBS (pH 7.4) and further fixed using 4% paraformaldehyde solution for 20 min. Further the cells were washed 3 times with 1X buffer with gentle agitation for 5 min and stored at −80°C prior to imaging. Two-photon excitation fluorescence imaging with linear confocal channel was used to visualize the uptake of nanogels by primary microglial cells. The laser scanning imaging system was custom built on Thorlabs Cerna microscope chassis (Thorlabs Inc., Newton, NJ, United States) with broadband femtosecond Ti:Sapphire laser (800 nm central wavelength, 85 MHz repetition rate, Element 600, Femtolasers, Vienna, Austria) as an excitation/illumination source. Confocal linear reflectance and TPEF images were acquired reconstructed using data acquisition board (NI PCIe-6351, Austin, TX, United States) from photomultiplier tube detectors (PMT, Hamamatsu, Japan) signals with suitable optical bandpass filters (775–785 nm for confocal; 465–495 nm and 550–633 nm for TPEF, Semrock, Rochester, NY, United States) at 1.33 frames per second by averaging 120 frames. PMT control voltages for the respective acquisition channels were kept constant between imaging sessions of control and treated samples, while the average laser power was adjusted in the range from 5 to 20 mW. All the acquired data in confocal and TPEF channels were normalized to a maximum value between nanogels treated and control (untreated) samples, final images depict square root of the intensity for visualization purposes. The concentration of nanogel (50 μg/ml) was selected based on the other experiments (flow cytometry) as the optimum concentration to see the time based uptake by the primary microglial cells.

#### Biodegradation Studies

The hydrolytic and enzymatic degradation of the hydrogel particles was studied using SEM analysis. Nanogel of known concentration 1 mg/ml was suspended in water for 7 days at 37°C. Cellulase, Trichoderma viride, was used for enzymatic degradation. Nanogels (1 mg/ml) were incubated for 7 days containing 10 units/g in 1 ml of water (pH 5.0) and then incubated particles from both the solution water and enzyme were dried on a glass slide and further coated with gold particles followed by SEM analysis to assess the morphology of the degraded particles.

#### Nanogel Transmigration Across the *in vitro* Blood Brain Barrier (BBB)

The *in vitro* BBB system used in the present study was adapted from previous studies published by Persidsky et al. (1997). Briefly, using a 24-well transwell (3.0 μM pore; Corning Life sciences) plate primary human brain microvascular endothelial cells (HBMEC; Sciencell) (2 × 105 per/well) were cultured in the inner chamber (on the upper membrane face) while primary human astrocytes (HA) and pericytes (at 1:1 ratio) were seeded (2 × 105 per well) on the lower membrane. These cells all together form the *in vitro* BBB transwell system which was co-cultured to confluency (∼7 days) prior to the treatment with different concentrations of nanogels.

##### Transendothelial electrical resistance (TEER) measurements.

The confirmation of BBB integrity was done by the measurement of transendothelial electrical resistance (TEER). The various nanogel concentrations were incubated for 24 h and the electrical resistance across the *in vitro* BBB was measured using an automatic ohmmeter (AutoRems, WPI). Briefly, BBB transwell system was placed on the AutoRem device, and the probes were placed in the inner and outer chamber. The TEER reading was measured at 15 s intervals. Results were normalized to the control/untreated wells.

##### Dextran-FITC transport assay for permeability assessment.

The permeability of the BBB was assessed using a Dextran-FITC transport assay. Briefly, Dextran-FITC solution (50 μl, 2 mg/ml) was added to the inner chamber of BBB transwell and the plate was incubated at 37°C for 3 h. Fluorescence (485/520 nm) in outer wells after 3 h was measured using microplate reader (Synergy HT, multi-mode microplate reader, Biotek). The percentage transport of FITC- dextran transport across the BBB model was compared with the FITC-dextran transported across the inserts without cells (Jayant et al., 2015). Results were normalized to the cell control/untreated post-incubation.

##### Nanoformulation transmigration efficiency measurement.

Transmigration study was carried out by using the auto-fluorescence feature exhibited by the nanogel particles. The nanogel in various concentrations was added to the upper chamber and incubated for 24 h. After the incubation of the media containing the nanogels was collected from the upper as well as the lower chamber. The fluorescence intensity was observed on a plate reader (Synergy HT, multimode microplate reader, BioTek) by adjusting the excitation and emission wavelength to excitation 590/20, emission 645/40.

Transmigration efficiency is measured as:

$$\%\mathrm{transmigration}\mathrm{efficiency}\;=\frac{\mathrm{Fluorescence}\,\mathrm{in}\,\mathrm{lower}\,\mathrm{chamber}}{\begin{array}{c} \mathrm{Fluorescence}\,\mathrm{in}\,\mathrm{upper}\,\mathrm{chamber}\\ +\mathrm{Fluorescence}\,\mathrm{in}\,\mathrm{lower}\,\mathrm{chamber} \end{array}}\times 100$$

The effect of the nanogel exposure on the integrity and permeability of the *in vitro* BBB was determined by measuring the TEER and the FITC dextran transmigration, respectively, using the Millicell ERS microelectrodes (Millipore). Briefly, FITC- dextran was added to the upper chamber and incubated for 4 h. Fluorescence was measured at ex/em 485/520 nm using a microplate reader (Synergy HT, multi-mode microplate reader, BioTek). The percentage of FITC- dextran transported across the BBB was compared with the FITC-dextran transported across the inserts without cells.

### Statistical Analysis

Data were represented as mean ± SD of three independent experiments or otherwise indicated. *T*-test was used for statistical comparisons among two groups. For statistical comparisons of more than two groups, one-way ANOVA was used to analyze the significant differences and wherever appropriate *p*-values of less than 0.03 were considered statistically significant. All the experiments were conducted in triplicates and results were expressed as mean ± standard deviation. Other statistical analysis are mentioned with individual figure captions.

## Results

### Design and Synthesis of Micro- and Nanogels Based Carriers

**FIGURE 1:**
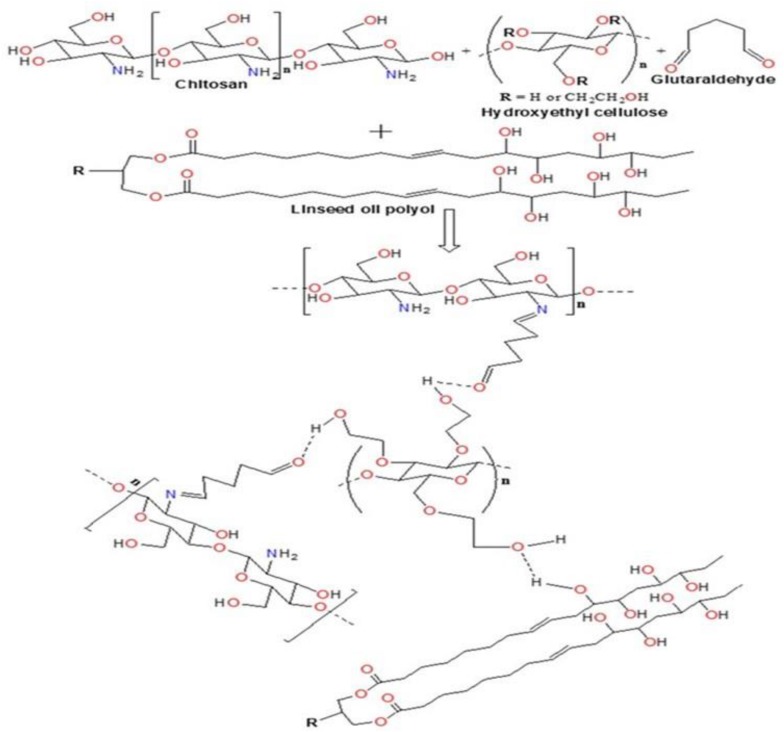
Reaction scheme for the development of micro-and nanogels.

**FIGURE 2:**
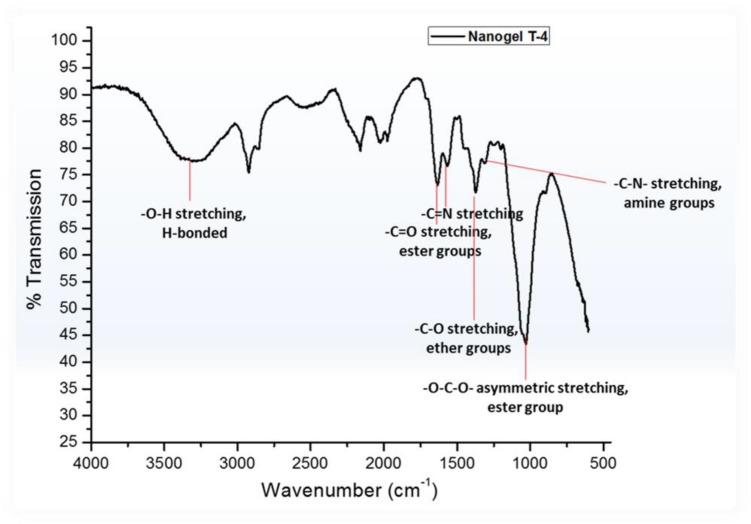
FT-IR analysis of nanogel.

Efforts have been made to design various hydrogel-based carriers using biopolymers owing to their superior characteristics with no toxicity and complete biodegradability. In the present study, biopolymers chitosan having medium molecular weights (190–310 kDa) and HEC have been used for the development of hydrogel particles. The water-in-oil emulsion polymerization reaction was deployed for the development of hydrogel particles. These hydrogel particles were hydrophobically modified using sustainable resource “Linseed oil” based polyol (Vashist et al., 2013). It was hypothesized that the hydrophobic modification will restrict the swelling capacity of the polymeric hydrogel particles, which thereby will enhance their hydrophobicity and thus help them traverse through the BBB. The hydrogel particles can be designed in various size ranging from micro to nano (300 μm to 100 nm). Linseed oil-based polyol was synthesized as per protocol reported earlier with some modification (Sharmin et al., 2007). The modulation in the stirring rate 1200–1400 rpm resulted in the development of finer and homogeneous hydrogel particles coded as T-4 in all figures. The reaction time played a crucial role in achieving the high yield of the hydrogel particles. Most importantly, it is worth to mention that there was no crosslinked particle formation when only HEC and polyol was used for the synthesis of nanogel by emulsion polymerization technique. Thus it is very important to highlight that chitosan played a significant role in the formation of crosslinked hydrogel particles. Previously, we reported the formation of inter-penetrating network with the addition of linseed oil derived polyol (Vashist et al., 2012). The concentration of the linseed oil-based polyol was found to modulate the size of the hydrogel particle and a decrease in particle size was observed with the increasing content of polyol in the synthesized hydrogels. Size sorting strategy was opted to obtain different size particles. Briefly, different size mesh filters were used for collecting the desired size particles. Emphasis was given on obtaining both micro and nanoparticles with unique characteristic properties, which can be diversely used as a carrier for therapeutics in cancer, HIV, other neurological disorders and diagnostics. Finally, the particles filtered through 25 μm membrane were used for all downstream experiments. Figure 1 showed the crosslinking reaction of the matrix materials (chitosan and HEC with glutaraldehyde and the surface modification by hydrophobic polyol was further confirmed by FT-IR analysis (Figure 2).

**FIGURE 3:**
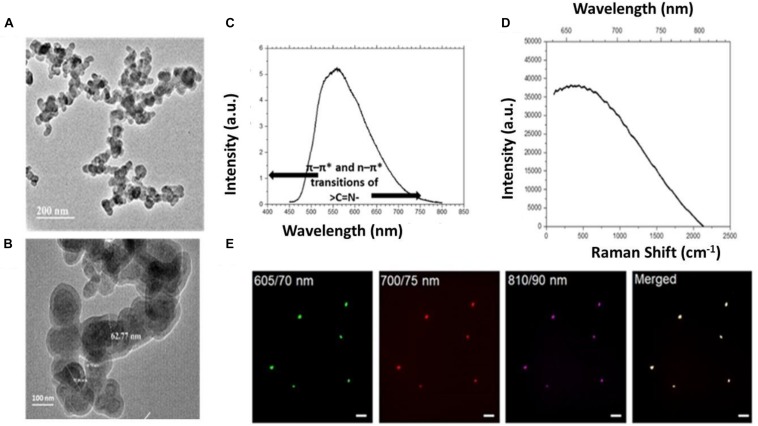
**(A,B)** TEM analysis for nanogel particles. **(C)** Photoluminescence (PL) measurements 405 nm diode laser as excitation source in Ocean Optics USB 2000 fiber optic spectrometer. **(D)** Raman spectra analysis of the nanogels. **(E)** Representative images showing the autofluorescence exhibited by the nanogels acquired at the wavelength regions (emission wavelength/band-width) for red (605/70 nm), far-red (700/75 nm) and near infra-red (810/90 nm) regions; an overlay of merged image is also shown, scale – 20 μm.

The core-shell structure was obtained by the transmission electron microscopy (TEM) (Figures 3A,B) for the designed hydrogel particles. TEM analysis confirmed the spherical shape with core-shell morphology and the nano-size (60–70 nm) of the particles. Supplementary Figure S1 shows the TEM image of single nanogel particles. The average hydrodynamic size was obtained using zeta sizer for the sorted particles (0.2 and 25 μm membranes) (Supplementary Figure S2) which showed that the sorting technique may be used to achieve the desirable hydrodynamic size of the developed micro/nanogel particles. The Zeta Potential measurements demonstrated the surface charge of −1.98 mV in water and −6.56 mV in PBS. It is expected that this near neutral surface charge will help in increased circulation time and also inhibition to plasma protein absorption to the surface of the nanogel particles.

### Fluorescence Characteristics of Micro- and Nanogel Particles

Apparently, the two individual biopolymers chitosan and HEC used in the present study for the design of hydrogel particles, show weak intrinsic fluorescence. Chitosan in aqueous solution, owing to its weak intrinsic fluorescence does not serve as a fluorescent probe to detect targets. Therefore, many strategies have been employed to conjugate fluorescent moieties to chitosan (Benediktsdóttir et al., 2012; Bor et al., 2017) and expand the horizon of its applications (Zaitsev et al., 2015; Bor et al., 2017). Chitosan-based polymers are usually tagged with fluorescent dyes/compounds for *in vitro* or *in vivo* biological imaging. Further, present synthesis involves hydrophobic modification using linseed oil-based polyol that was pre-stored for 190 days. Linseed oil shows fluorescence in the range of 600 nm when excited at 337 nm (Miyoshi, 1985; Alam et al., 2014). The confirmation of the auto-fluorescence was done using photoluminescence (PL) measurements, which was conducted using Ocean Optics USB 2000 fiber optic spectrometer and 5 mW, 405 nm diode laser as the excitation source (Figure 3C). Interestingly the Raman spectral analysis, which revealed the presence of the strong autofluorescence in the micro- and nanoparticles (Figure 3D). Strong fluorescence is known to muddle the weak spontaneous Raman signals which add to challenges in chemical characterization of single particles (Kaiser et al., 1996; Gong et al., 2017). Therefore, the absence of Raman peaks in the Raman spectra was intriguing and pointed toward the presence of high fluorescence exhibited by the particles (Figure 3D). To further validate the efficacy of the developed hydrogel particles for *ex vivo* or *in vivo* imaging, the particles were imaged at higher wavelengths using optical filters (emission wavelength/band-width) for red (605/70 nm), far-red (700/75 nm) and near infra-red (810/90 nm) regions. The particles showed a bright emission at all the wavelengths including 810 nm (Figure 3E).

Additionally, images were acquired using imaging flow cytometer. Different concentrations (1–100 μg/ml) of nanogel particles without cells were prepared in PBS buffer pH 7.4 to get an emission spectrum of the particles at an excitation of 405 nm and confirmed the presence of fluorescence. Post-acquisition of nanogel particles, analysis was done based on the area and aspect ratio of images, single-particle population and clumps were gated. A concentration-dependent reduction in the fluorescence of diluted samples was observed, which may be attributed to the less availability of single particles on dilutions.

**FIGURE 4:**
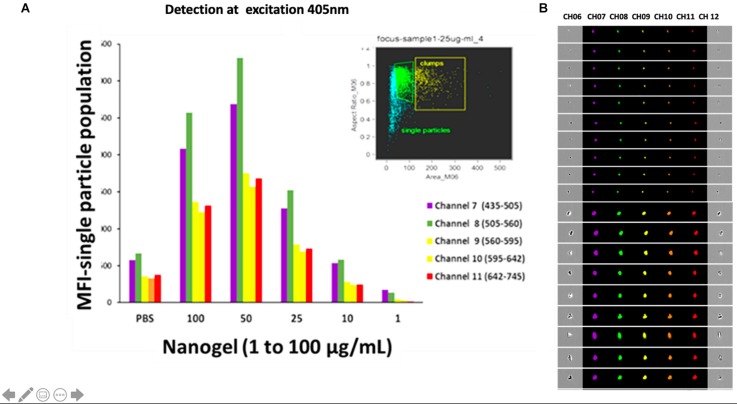
**(A)** The mean intensity values of the single-particle population for each channel at an excitation wavelength of 405 nm. **(B)** Images acquired for the presence of fluorescence for and aggregated and single particle in each channel at an excitation wavelength of 405 nm.

Figure 4A represents the mean fluorescent intensity values of the single-particle population for each channel. The nanogel particles were detected with 405 nm excitation laser and analyzed based on emission in channel 8 (505–560 nm), which exerted the maximum intensity for each concentration. 50 μg/ml nanogel concentration gave the maximum fluorescent intensity (Figure 4B). Our results clearly indicate that the polymeric network particles formed by the reaction of chitosan, glutaraldehyde, HEC, and linseed polyol showed wide emission range (460–770 nm) when excited at 405 nm. The crosslinking reaction between chitosan and glutaraldehyde possibly led to the formation of Schiff’s bases by condensation reaction between the amino and carbonyl groups of chitosan and glutaraldehyde, respectively. HEC and linseed polyol are stable in the polymeric network due to the formation of hydrogen bonds. Overall, in the polymeric network, as shown in the reaction scheme in Figure 1, we have different types of functional groups that may have enhanced the fluorescing ability of the hydrogel network. As discussed in the earlier section it is important to highlight that chitosan was the most important constituent in combination with HEC and polyol, which played a crucial role for the presence of high fluorescence in the developed particles. The additive effect of the –OH groups (due to the incorporation of polyol) and the formation of the > C = N- linkages (due to condensation reaction between chitosan and glutaraldehyde) may have enhanced the fluorescing ability of the polymeric network in the wavelength range of 430–630 nm. Overall, the attachment of the linseed polyol containing multiple –OH groups may have contributed largely to the fluorescing capacity of the hydrogel network (460–770 nm). The electronic transitions of particular interest for fluorescence in the reported polymeric network are the low energy π–π^∗^ and n–π^∗^ transitions of > C = N, and high energy n-σ^∗^ transitions of –OH groups. The broadening of the peak, when excited at 405 nm, which is centered at 570 nm, may be attributed to the coalescing of the fluorescence bands of the peaks due to –OH groups with slightly different energies.

### Biocompatibility Evaluation of Nanogels *in vitro*

**FIGURE 5:**
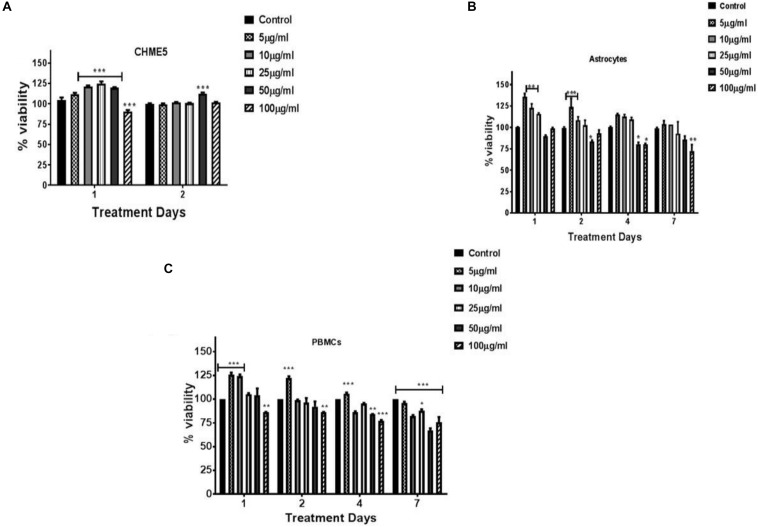
Cytocompatibility testing of nanogels at various concentrations (5–100 μg/ml) as a function of time. **(A)** CHME-5; **(B)** astrocytes; **(C)** PBMCs. Cells were treated with various concentration of nanogel for 1, 2, 4, and/or 7 days. XTT assay performed on cells post nanogel exposure. Statistical significance determined by Two-way ANOVA and *post hoc* (Bonferroni post-tests) analysis, *p < 0.05, **p < 0.01, and ***p < 0.001.

To evaluate the biocompatibility of the synthesized nanogels, LDH assay (was performed on neuronal cells Microglia and PBMCs (Supplementary Figure S3). Results demonstrated that the nanogels were non-cytotoxic and the viability was similar to the control and thus are safe to the cells. Cytotoxicity of nanogels against the peripheral cells (PBMCs), astrocytes and microglial cells (CHME5) was assessed by XTT assay performed over a concentration range of 10–100 μg/ml. The nanogels were found to be non-toxic in the tested concentrations up to 2 days for CHME5. The high cell viability (∼80% and above up to 7 days) and intact morphology confirmed that the developed nanogels are biocompatible with respect to all the cell types tested (Figure 5). This may be attributed to the biocompatible biopolymers used for the development of the hydrogel particles and their unique chemical structure resulted after crosslinking, exhibiting biomimetic soft tissue like hydrogel structures. Also, we hypothesize that the enhanced element of hydrophobicity and formation of interpenetrating networks (IPNs) introduced by incorporation of linseed oil-based polyol may result in a stable nanogel system which sustained in the cellular environment.

### Toxicity Analysis of Nanogels in Human Astrocytes

**FIGURE 6:**
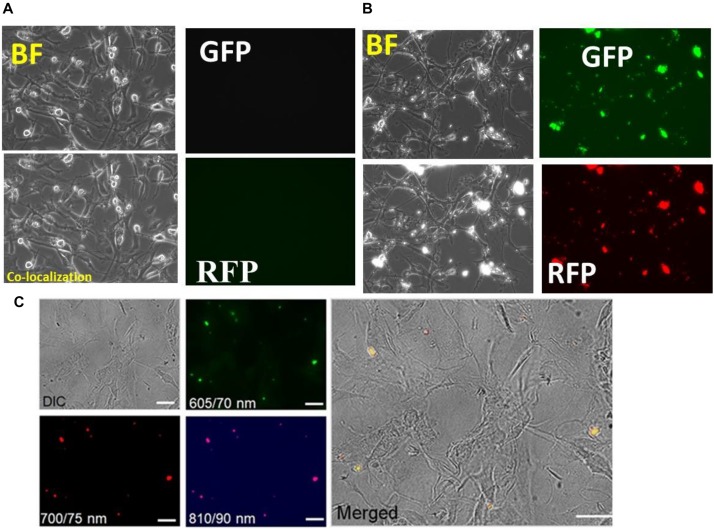
Toxicity profiles and co-localization of nanogels with astrocytes after 7 day treatment of nanogels. **(A)** Control; **(B)** 100 *upmu*g/ml. **(C)** Cellular uptake analysis of the nanogels as assessed by the differential interference contrast (DIC) imaging and by the fluorescent imaging at the wavelength/bandwidth range of 605/70, 700/75, and 810/90 nm, scale – 20 μm. The merged image on the right panel shows the overlay of all the images.

Human astrocyte cell line (U87 cells) were cultured for 24 h and were incubated with nanogel particles suspended in PBS at a final concentration of 100 μg/ml. Images were acquired at day 6 (Supplementary Figure S4) and day 7 using various optical filters for analyzing fluorescence in the green, red and blue visible region (Figures 6A,B) in OLYMPUS IX51 microscopy system. There were no discernible morphological changes in the astrocytes incubated with nanogels compared to untreated cells; therefore, nanogels were deemed as non-toxic for this CNS cells. The longer treatment of astrocytes with nanogels demonstrated cellular uptake of nanogels suggesting longer retention (up to 7 days) of these particles within astrocytes; therefore, confirming the potential use of these nanogels for CNS-targeted theranostics.

Cellular co-localization of particles within astrocytes was demonstrated by imaging the cells that were incubated with the 10 μg/ml of nanogels for 72 h in the Labtech chambered slides at different emission wavelengths (CWL)/Bandwidth (FWHM): 605/70, 700/75, and 810/90 (Figure 6C). A differential interference contrast (DIC) image showed the cell morphology, and the merged image analysis with fluorescent images showed the co-localization of the particles within the cells and particles were imaged using the higher wavelength filters. These studies further confirmed the potential for *in vitro* and *in vivo* imaging utility of these novel nanogel particles as they can be imaged in the far-red and near-infrared regions for better imaging penetrance.

### Cell Uptake and Toxicity Profiles

In addition to the biocompatibility testing, the nanogels were qualitatively and quantitatively screened for the cellular uptake using single-cell imaging flow cytometry. For this study, CHME5 cell lines and PBMCs were selected. As discussed in the earlier section, different concentrations of nanogels were incubated with cells for up to 24 h. After incubation, cells were harvested, washed with PBS, acquired, and analyzed to quantify the location and distribution of fluorescence. In this process of analysis, we further confirmed the existence of wide emission wavelength and multichannel fluorescence properties of nanogels. The cells treated with nanogel particles were detected with 405 nm excitation laser and analyzed based on emission in channel 8 (505–560 nm), which exerted the maximum intensity for each concentration.

**FIGURE 7:**
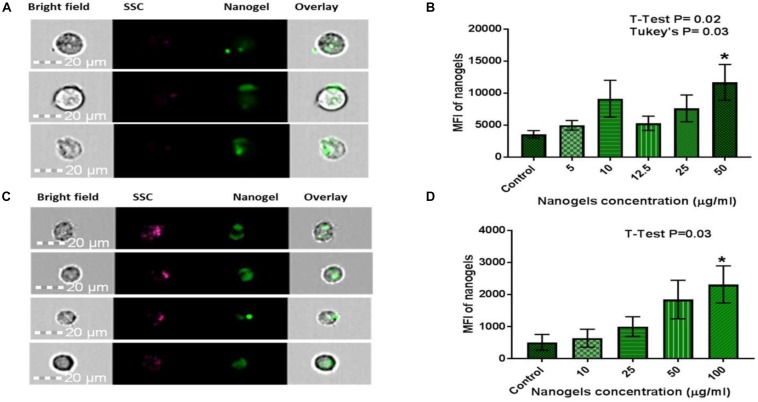
Qualitative and quantitative assessment of cellular uptake of nanogels using Flow Cytometry for **(A,B)** CHME-5: Statistical analysis: Data is represented as Mean ± SEM. Significance was tested by Student’s *T*-test which showed T4 50 μg/ml was significantly higher than Control (*P* = 0.02). Significance was also tested by one-way ANOVA which showed significance between columns (treatments), *F*(5, 28) = 2.786, *P* = 0.0365. *Post hoc* analysis with Tukey’s multiple comparisons tests showed T4 50 μg/ml was significantly higher than control (*P* = 0.03). **(C,D)** PBMCs: Statistical analysis: Data is represented as Mean ± SEM. Significance was tested by Student’s *T*-test which showed T4 100 μg/ml was significantly higher than Control (*P* = 0.03). Significance was also tested by one-way ANOVA which showed significance between columns (treatments), *F*(4, 23) = 2.995, *P* = 0.03. *Post hoc* analysis with Tukey’s multiple comparisons tests did not show any further significance. * means it is significant compared to control.

Time and concentration dependent uptake of nanogels by PBMCs and CHME5 (1 × 10^6^) was confirmed using imaging flow cytometry. PBMCs cultured with nanogels at a concentration of 100 μg/ml showed significant uptake of nanogel particles. Similarly, CHME5 cells, showed significant uptake at 50 μg/ml concentration. Images acquired for CHME5 expressing fluorescence due to nanogel uptake and the mean fluorescent intensity values of the positive cell population are shown in Figures 7A,B. Similarly, Figures 7C,D demonstrate the percentage of PBMCs expressing fluorescence due to nanogel uptake and the mean fluorescent intensity values of the positive cell population. A time-dependent increased in fluorecence of cells cultured with nanogels for 2 and 6 h is demonstrated in bar graphs included in Supplementary Figure S5. The further increased in fluorecence after culturing cells with nanogels for up to 24 h (Supplementary Figure S5) confirmed a time-dependent increase in cellular internalization of nanogels.

### Two-Photon Imaging

**FIGURE 8:**
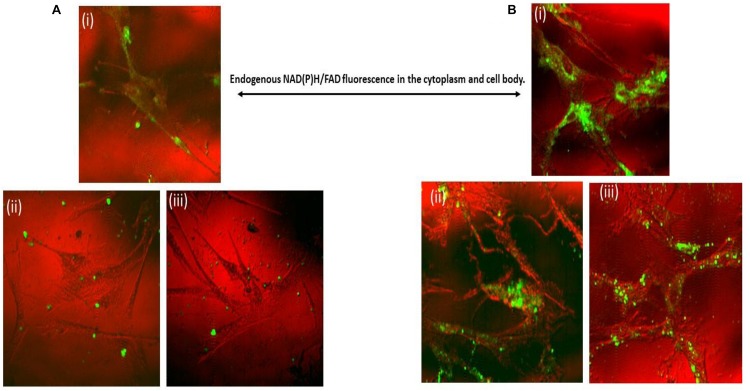
Combined reflectance confocal and two-photon imaging of nanogel uptake by Primary microglial cells at **(A)**: (i) Control; (ii,iii) 6 h treatment; **(B)**: (i) Control; (ii, iii) 24 h treatment of nanogels. Pseudocoloring: Red – confocal (or nearly confocal) reflectance at 780 nm; Green – Two-photon excitation fluorescence (400–633 nm range all together). Average laser power ranging from 5 to 20 mW.

The two-photon fluorescence imaging also confirmed the cellular uptake of nanogels for up to 24 h. Figure 8 showed the 6 and 24 h time based uptake of nanogel by primary microglial cells. Control (untreated) and nanogel treated cells were imaged and are depicted in Figure 8. The time based uptake showed that there was increased uptake of nanogels in 24 h. Please note that the untreated cells have background endogenous fluorescence (Figure 8), which is due to the presence of NADH/FAD fluorescent co-factors in the cytoplasm and cell body. The imaging technique showed the presence of fluorescent nanogel particle in primary microglial cells in 465–495 nm and 550–633 nm channels.

### Hydrolytic Degradation and Enzymatic Degradation Study

The nanogel were found to change little in morphology after 7 days of hydrolytic (water) and enzymatic degradation (Cellulase) study as indicated by the SEM analysis (Supplementary Figure S6). The hydrolysis and complete degradation of nanogels in 7 days was limited due to the stability achieved by the crosslinking and the hydrophobicity induced by the addition of linseed polyol. Though, it is expected that longer incubation time of the nanogel will induce complete biodegradation of these nanogels due to the biodegradable characteristic of chitosan (Wang et al., 2019) and HEC which is used as the matrix material in the development of hydrogels.

### Blood-Brain Barrier (BBB) Transmigration

**FIGURE 9:**
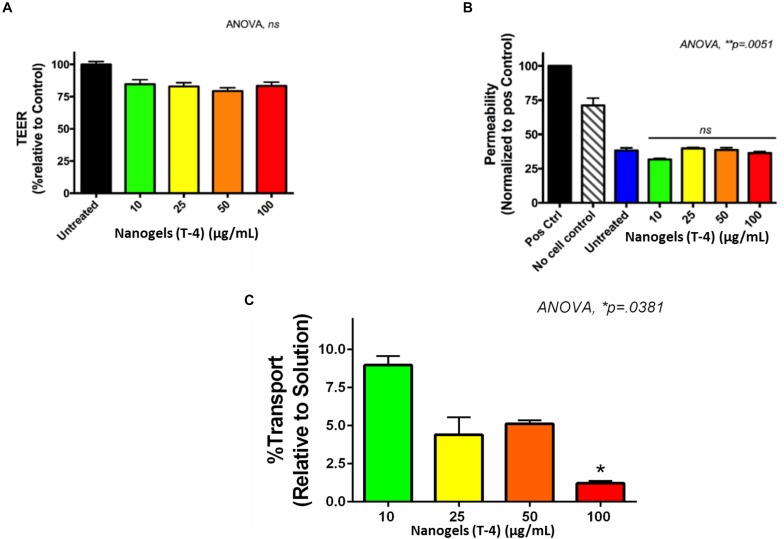
*In vitro*- BBB model. **(A)** The TEER values of the *in vitro* BBB model after exposure of nanogels. **(B)** Permeability of the *in vitro* BBB model after nanogel treatment for 24 h. **(C)** % transport of nanogels across the *in vitro* BBB model.

The BBB poses a great challenge for efficient delivery of various therapeutics owing to its lipid-rich composition (Hawkins et al., 2006). This necessitates the requirement of formulations which provides a hydrophobic environment for ease of transmigration through BBB. Therefore the transmigration of nanogels through the BBB was evaluated using the *in vitro* human BBB model. The intactness of the developed BBB was determined by measurement of TEER values. As the integrity of the BBB was confirmed the nanogels in different concentration (10–100 μg/ml) were added to the different upper wells of BBB. Figure 9A shows the TEER values of the control and the treated wells of various concentration of nanogels. There was no significant change in the values of TEER as compared to the control, which confirms that the nanogels does not affect the overall integrity of the BBB. The permeability of the BBB was also measured using the paracellular transport of the FI-TC across the BBB as described previously (Jayant et al., 2015). The FI-TC is used as a detection bioactive molecule for the membrane intactness. Figure 9B shows that the permeability was not altered with the treatment of nanogels as compared to untreated. The permeability is normalized to the positive control.

The transmigration of the nanogels was measured as the function of fluorescence. The fluorescence intensity recorded in upper and lower chambers using plate reader showed that approximately 10% of nanogels were capable of transmigrating through the tough BBB tight junctions (Figure 9C). The hydrogel particles used in this study were polydispersity with PDI of 0.6. Hydrophobically modified nanogels using linseed oil (triglyceride) based polyol which is non-polar in nature have been designed in such a manner which increases their hydrophobicity for enhancing their transmigration across the tight junctions. The crosslinking reaction occurred during the synthesis of nanogels between the biopolymers viz., chitosan, HEC and glutaraldehyde leading to the formation of Schiff base using the condensation reaction and the surface functionalization by the linseed polyol may have facilitated hydrophobic interactions. The transport data revealed that unlike at higher concentration (100 μg/ml), gel particles exhibited better transmigration in BBB model when used at lower concentration of 10 μg/ml. This may be possibly due to the less aggregation and less crowding of the nanogel particles in the upper chamber which may have allowed better diffusion across the tight junctions. The main cause of the lower transmigration of nanogels across the BBB may be due to the polydispersity of the particles and it is anticipated that there may be an increase in the % transport once the polydispersity is lowered down and a monodisperse particle with PDI of >0.4 is used. We hypothesize that slight variation in the content of the polyol, increasing the monodispersity, and the biopolymers may increase the passage of the nanogel through the tight junctions. Thus, these novel auto fluorescent nanogels owe great potential as a DDS for CNS therapy.

## Discussion

In recent years, several nano- and micron range drug delivery vehicles have evolved for theranostic applications remarkably due to rise in the demand for multi-functional systems capable of imaging and delivering the drugs or genes to the target site (Shi et al., 2014; Chan and Almutairi, 2016). For example, various nanoparticles based systems have been developed by conjugating with lanthanides, peptides, gold nanoparticles, and quantum dots which contributes to the fluorescence and can also act as a drug delivery vehicle (Lin et al., 2017; Cardoso Dos Santos et al., 2018; Yuan et al., 2018). However, these materials have faced a lot of safety concerns owing to toxicity due to their inorganic nature. Efforts are being made to develop safe autofluorescent biomaterials such as polymers (Yang J. et al., 2009), polymeric nanoparticles (Yang Z. et al., 2009), dendrimers (Wang and Imae, 2004), and nanogels (Gyawali et al., 2018) with significant easy route and high biocompatibility and less toxicity which can be deployed for bio-imaging and drug delivery (Vashist et al., 2018b). Our approach utilizes the natural biodegradable and biocompatible polymers and uses a simple method of synthesis developing both micro-and nano range particles exhibiting autofluorescence which could be a promising theranostic system that will mimic a perfect imaging agent and delivery vehicle for CNS drug delivery. The ability of the developed hydrogel particles to fluoresce in wide emission spectrum range and cellular compatibility could potentially translate the present imaging modalities and develop soft nanogel systems for CNS and periphery therapies.

In the present study, we have put forward the advanced and novel bio-polymeric hydrogel system in micro- and nanoscale exhibiting unique autofluorescent feature. This hydrogel system has wide-emission spectrum in the range of 450–780 nm, on excitation at 405 nm as well as excitation at 710 nm gives emission at 810 nm showing its capacity to be used for *in vivo* imaging. Recent trends in theranostics suggest that this wide emission phenomenon from a bio-polymeric biocompatible system offer excellent potential for bright fluorescence probes used for *in vitro* optical imaging used for cellular imaging as well as *in vivo* imaging (Anilkumar et al., 2013).

We showed that the developed nanogel show safe uptake by the microglia and PBMCs. These gel particles owing to their biodegradable and biocompatible constituents showed low cytotoxicity and high biocompatibility with all the cell types viz., PBMCs, CHME5, and astrocytes. The % viability of ∼80% using nanoformulations is considered to be non-toxic in both peripheral and CNS cells (Thorat et al., 2014). The toxicity investigation of the nanogels using the astrocytes long co-culture experiments revealed that the developed nanogel particles were biocompatible with the astrocytes for 7 days and there was co-localization of the nanogel particles with the astrocytes. The revelations of the autofluorescence by the Raman and photoluminescence study was further confirmed by the images acquired by the flow cytometry. This mechanistic study used for imaging nanogel provided proof of the concept to identify the cellular internalization and presence of fluorescence. This study further confirmed the potential application of the developed hydrogel particles in bio-therapeutic delivery by cellular co-localization or internalization (Li et al., 2017).

The developed micro- and nanogel system put forward their advantages as a multi-functional carrier escaping all the limitation offered by the conventional nanocarriers such as tagging with inorganic fluorescent probes, non-toxicity and another adverse side-effects. The exquisiteness of the present system lies in the wide-detection range of the nanogels as multi-channel fluorescence offer the benefit of the detection of the nanogels in various instruments by adjusting the excitation and emission wavelengths. We could observe the fluorescence on a plate reader (Synergy HT, multimode microplate reader, BioTek) by adjusting the excitation and emission wavelength to excitation 590/20, emission 645/40. This makes the “micro- and nanogel” a unique imaging agent with wide-detection limit and makes it accessible for various instruments. Moreover, the micro- and nanogels do not contain any drug and inorganic nanoparticles imply the presence of autofluorescence and biocompatibility, thus they have the potential to be used as an imaging agent for other bioactive molecules when binding with the developed nanogels.

Radically improved features of the present hydrogel system have advantages over the so far published fluorescent hydrogel systems in the literature (Chang et al., 2009; Ji et al., 2019; Wu et al., 2019). The BBB model explicitly showed the experimental proof for the hypothesized concept of the hydrophobically modified nanogels using linseed oil-based polyol transmigrating across the tight junctions of BBB. The data on the intactness of BBB and permeability after the exposure of the nanogels intriguingly suggest that the designed nanogel system owe great potential of CNS drug delivery. However, the exact mechanism of the transport of these nanogels crossing the BBB has to be explored in depth.

This study provides a room to develop a receptor or ligand-free delivery of nanocarrier for CNS drug delivery. Notably, present multi-functional, size-controlled nanogel showed high fluorescence and biocompatibility and exploits all size range from micro-and nanogels. The future direction of the present study directs for the improvement in the% transport of nanogels across BBB. The intonation in the concentrations of the biopolymers used in the present synthesis, the change in the concentration of hydrophobic polyol including variation in the reaction conditions such as stirring rate and temperature may be utilized to fabricate a new class of nanogels with improved features, i.e., enhanced transmigration through BBB. Thus, we propose these multi-functional nanogel particles as an innovative and effective tool for the CNS targeting and innocuous therapies and owe huge potential to act as an excellent theranostic agent.

## Conclusion

In summary, we here developed a novel bio-polymeric size-controlled approach for synthesizing micro- and nanogel particles exhibiting unique autofluorescent characteristic, which obviates the requirement of additional tag for their detection inside the intracellular microenvironment and exhibit potential for *in vivo* imaging. These hydrogel particles possess excellent biocompatibility, cell uptake, and surface functionality, which make them superior therapeutic carrier which can be explored for their capability to encapsulate various bioactives inside them. It is anticipated that both the hydrophobic modification by using linseed oil-based polyol and soft porous structure has facilitated the transport the hydrogel particles through the strong CNS BBB. This hydrogel systems strongly directs the technology toward the path of advancement in nanoimaging and therapy, which will provide new avenues for enhanced CNS therapy.

## Data Availability Statement

All datasets generated for this study are included in the article/Supplementary Material.

## Author Contributions

ArV planned and coordinated research and was actively involved in all experiments. AtV, AK, and AT helped in data analysis and manuscript writing. TP, MA, and AD helped in the detection of fluorescence by laser and cell uptake studies using flow cytometry measurements. HC contributed the microscopic imaging. VA and ZH performed cytotoxicity studies. RN-M was involved in TEM analysis. AR was involved in the design of BBB *in vitro* model and transport study. IS and JR-R helped us in two-photon imaging of the nanogels. MN was involved in the experimental research plan and continuous supervision.

## Conflict of Interest

ArV, MN, and AK have the following competing interest: Florida International University has a US Non-Provisional patent application entitled “Micro/Nano Magnetic Hydrogels with Autofluorescence for Therapeutic and Diagnostic Applications. This work has been granted the U.S. Patent on Micro/Nano Magnetic Hydrogels with Autofluorescence for Therapeutic and Diagnostic Applications US Patent App. 15/907, 703, 2019 with ArV, MN, and AK; as inventors. The remaining authors declare that the research was conducted in the absence of any commercial or financial relationships that could be construed as a potential conflict of interest.
