# Supplementary material for: Development of Multifunctional Biopolymeric Auto-Fluorescent Micro- and Nanogels as a Platform for Biomedical Applications
Source: Front Bioeng Biotechnol. 2020 Apr 30;8:315. doi: 10.3389/fbioe.2020.00315 (PMC7203429; doi:10.3389/fbioe.2020.00315)
Supplement: Supplementary file 1 [file Data_Sheet_1.pdf]

Development of Multifunctional Biopolymeric Auto-Fluorescent Micro- and Nanogels as a Platform for Biomedical Applications

Arti Vashist[1], Venkata Atluri[1], Andrea Raymond[1], Ajeet Kaushik[1,2], Tiyash Parira[1], Zaohua Huang[1,3], Andriy Durygin[4,] Asahi Tomitaka[1], Roozbeh Nikkhah-Moshaie[1], Atul Vashist[5], Marisela Agudelo[1], Hitendra S. Chand[1], Ilyas Saytashev[6,7], Jessica C. Ramella-Roman[6,7] and Madhavan Nair[1*]

[1]
*Corresponding Author: nairm@fiu.edu

Keywords: Nanogels; Microgels; Theranostics; Nanomedicine; Biopolymers

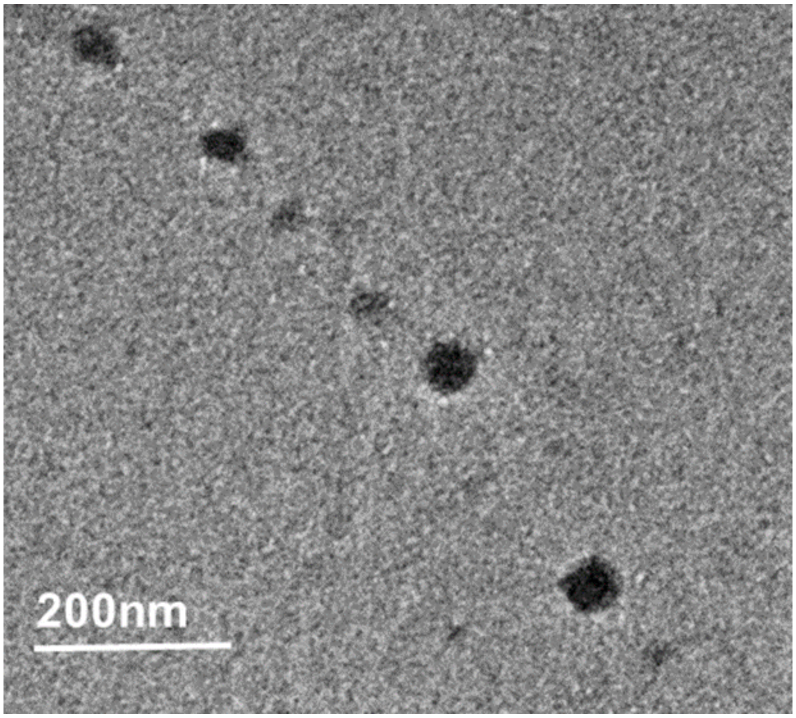

Figure S1 TEM analysis of nanogel particles.

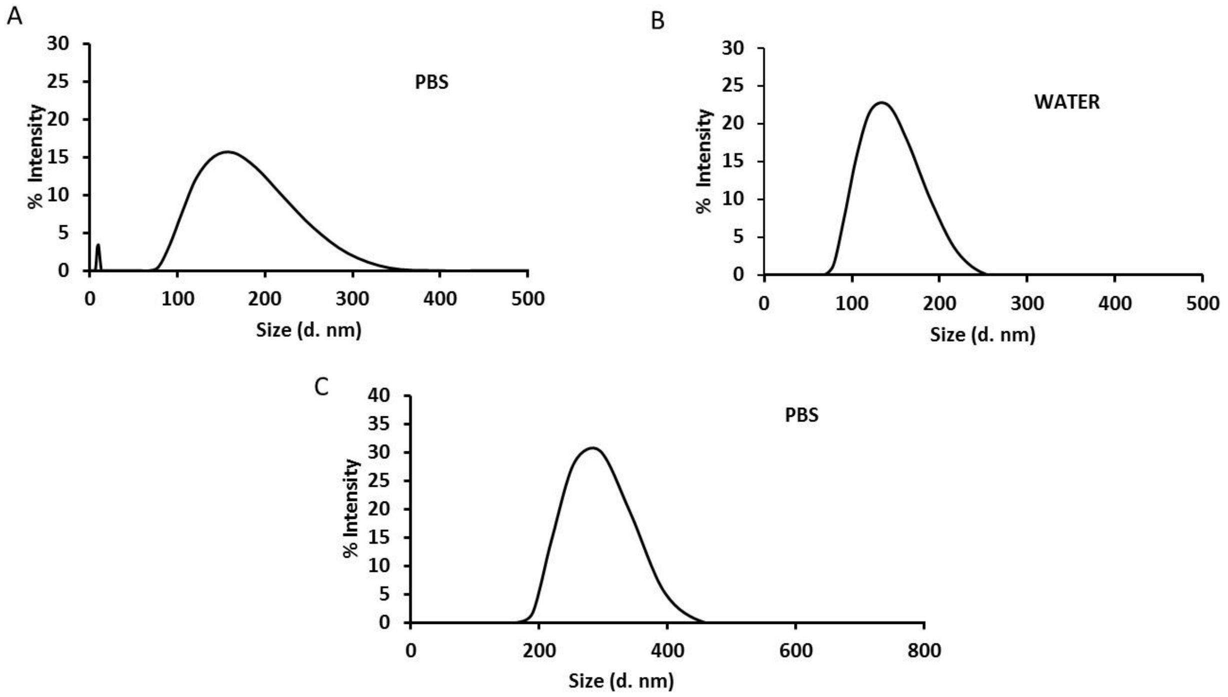

Figure S2 : Average Hydrodynamic size measurements of nanogel particles A) filtered through 0.2µm membrane PBS Z- average (d.nm): 130 nm; PdI= 0.410 ; B) filtered through 0.2µm membrane in water Z- average (d.nm): 127nm; PdI= 0.251 ; C) filtered through 25µm membrane Microgel particles in PBS Z- Average (d.nm): 533.9 nm ; PdI= 0.6.

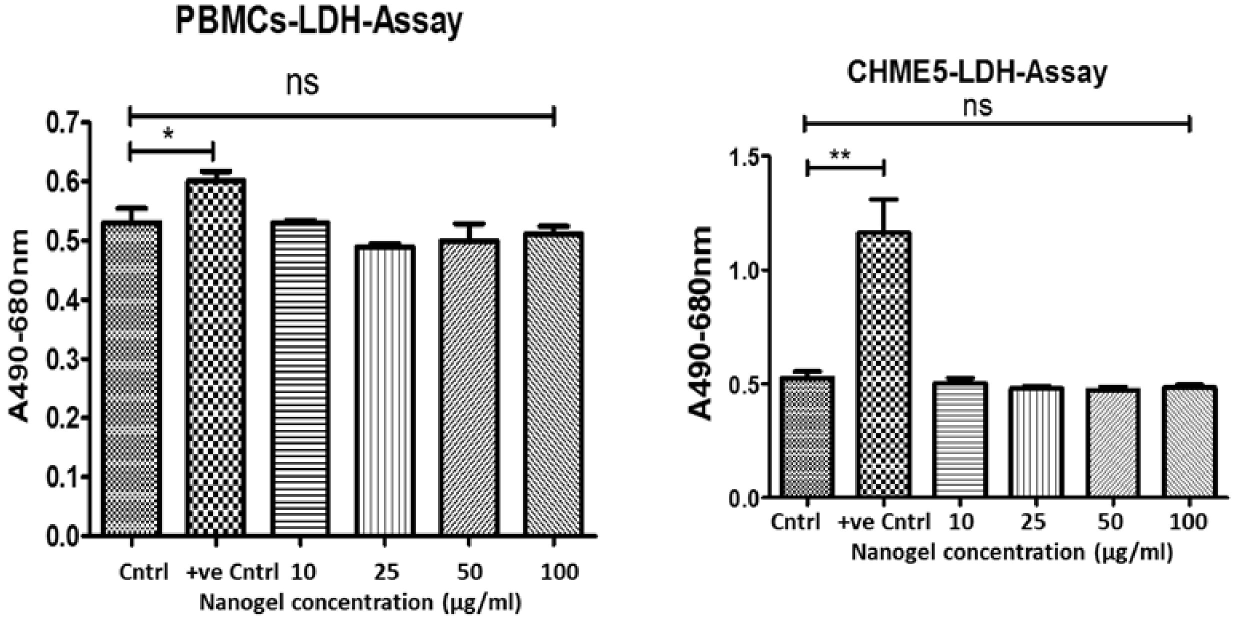

Figure S3 Lactate Dehydrogenase (LDH) cytotoxicity of Nanogel in Microglia (CHME5) and PBMCs

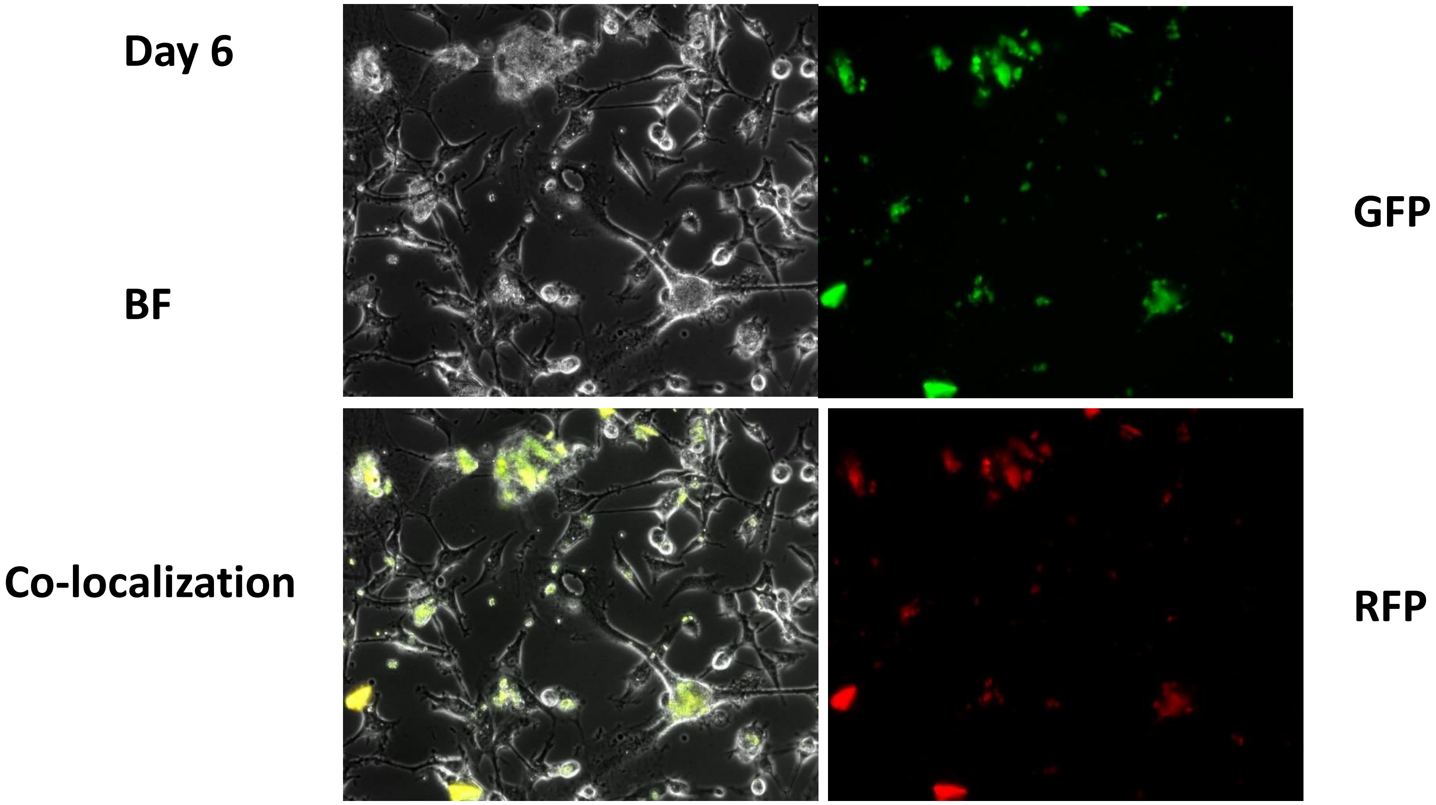

Figure S4 Images acquired for toxicity investigation and co-localization of nanogels (100μg/ml) with astrocytes on 6-day of long co-culture experiment.

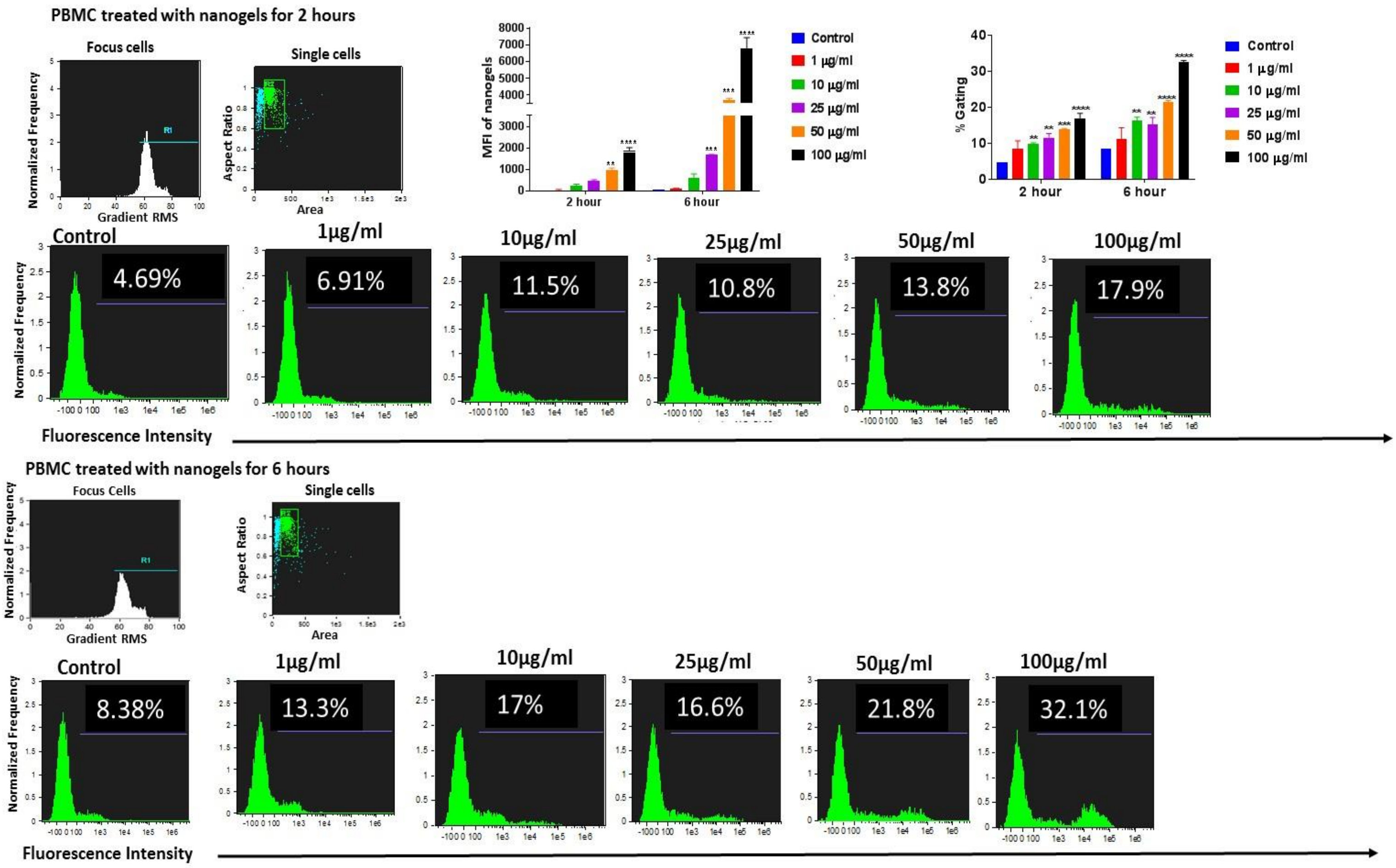

Figure S5 Time-based kinetics study of nanogel particles at different time point 2 hour and 6 hour with PBMCs.

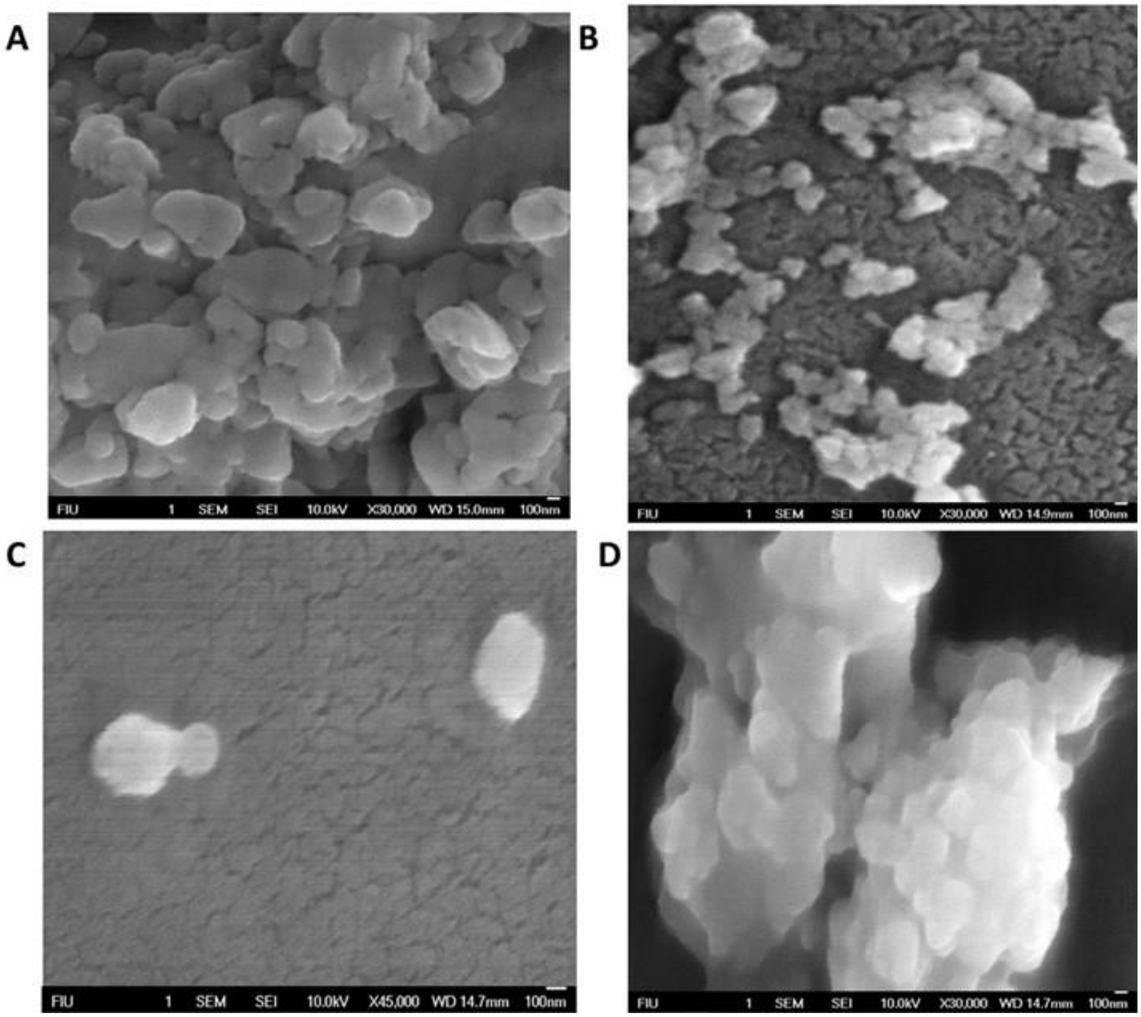

Figure S6 Hydrolytic degradation study in water for A) 24 hours; B) 7 days; Enzymatic (Cellulase: 10 units/ml ;pH 5) degradation in 7 days C) isolated nanogel particles; D) aggregated nanogel particles.
